# Decitabine assists umbilical cord-derived mesenchymal stem cells in improving glucose homeostasis by modulating macrophage polarization in type 2 diabetic mice

**DOI:** 10.1186/s13287-019-1338-2

**Published:** 2019-08-19

**Authors:** Jieqing Gao, Yu Cheng, Haojie Hao, Yaqi Yin, Jing Xue, Qi Zhang, Lin Li, Jiejie Liu, Zongyan Xie, Songyan Yu, Bing Li, Weidong Han, Yiming Mu

**Affiliations:** 1 Department of Endocrinology, Chinese PLA General Hospital, Medical School of Chinese PLA, Beijing, China; 2 Department of Endocrinology, Beijing Rehabilitation Hospital of Capital Medical University, Beijing, China; 3 Department of Molecular Biology, Institute of Basic Medicine, School of Life Science, Chinese PLA General Hospital, Beijing, China; 4 Department of Endocrinology, Beijing Tiantan Hospital, Capital Medical University, Beijing, China; 5 Department of Endocrinology, Chinese People’s Liberation Army General Hospital, Beijing, China; 6 Department of Geriatrics, China-Japan Friendship Hospital, Beijing, China

**Keywords:** Mesenchymal stem cells, Insulin resistance, Decitabine, Macrophage polarization, Diabetes

## Abstract

**Background:**

Mesenchymal stem cells (MSCs) have emerged as a promising therapy for type 2 diabetes (T2D). Mechanistic researches demonstrate that the anti-diabetic effect of MSCs is partially mediated by eliciting macrophages into an anti-inflammatory phenotype thus alleviating insulin resistance. However, single MSC infusion is insufficient to ameliorate sustained hyperglycemia or normalize blood glucose levels. In this study, we used decitabine (DAC), which is involved in the regulation of macrophage polarization, to test whether MSCs combined with decitabine can prolong and enhance the anti-diabetic effect in T2D mice.

**Methods:**

High-fat diet (HFD) and streptozocin (STZ) were given to induce T2D mouse model. Successfully induced T2D mice were randomly divided into four groups: T2D group, MSC group, DAC group, and MSC + DAC group. Blood glucose was monitored, and glucose tolerance and insulin sensitivity were evaluated during the entire analysis period. Epididymal fat was extracted for analysis of macrophage phenotype and inflammation in adipose tissue. In vitro, we examined the effect of MSC + DAC on macrophage polarization in bone marrow-derived macrophages (BMDMs) and explore the possible mechanism.

**Results:**

MSC infusion effectively improved insulin sensitivity and glucose homeostasis in T2D mice within 1 week, whereas combination therapy of MSCs + DAC extended the anti-diabetic effects of MSCs from 1 to 4 weeks (the end of the observation). Correspondingly, more M2 macrophages in adipose tissue were observed in the combination therapy group over the entire study period. In vitro, compared with the MSC group, MSCs combined with decitabine more effectively polarized M1 macrophages to M2 macrophages. Further analysis showed that the effect of MSC + DAC on macrophage polarization was largely abrogated by the peroxisome proliferator-activated receptor gamma (PPARγ) antagonist GW9662.

**Conclusions:**

Our data suggest that MSCs combined with decitabine can more effectively alleviate insulin resistance and prolong and enhance the anti-diabetic effect of MSCs in T2D mice in part by prompting M2 polarization in a PPARγ-dependent manner. Thus, decitabine may be an applicable addition to MSCs for diabetes therapy.

**Graphic Abstract:**

UC-MSCs combined with decitabine activate the IL4R/STAT6/STAT3/PPARγ axis to further promote M2 macrophage polarization in adipose tissue, reduce inflammation, improve insulin sensitivity, and lead to better glucose metabolism and long-term hypoglycemic effects 
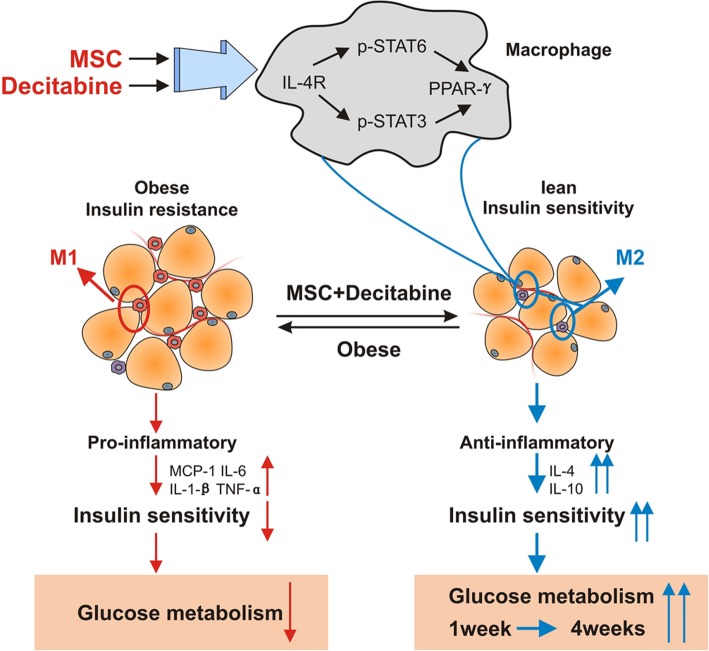

**Electronic supplementary material:**

The online version of this article (10.1186/s13287-019-1338-2) contains supplementary material, which is available to authorized users.

## Background

Type 2 diabetes (T2D) is a major public healthcare problem affecting over 400 million people worldwide [1]. Although various pharmacological agents have been applied to the treatment of T2D, fewer than 60% of patients achieve the goal of HbA1c < 7.0% [2, 3]. Insulin resistance, a major etiologic feature, plays a crucial role throughout the entire development of T2D [4]. Thus, attenuating insulin resistance has always been the foremost therapeutic goal. In clinical practice, metformin as well as thiazolidinedione (TZD) was used to improve insulin sensitivity, but up to 30% of patients cannot tolerate the troublesome gastrointestinal side effects of metformin [5]. In addition, the use of TZD has been curtailed in recent years due to its increased cardiovascular morbidity and potential toxicities [6, 7]. Therefore, the development of alternative therapies with novel mechanisms is needed to alleviate insulin resistance and help patients achieve better glycemic control.

Mesenchymal stem cells (MSCs) are a pluripotent cell population that can differentiate into multilineage cells, modulate the local environment, and regulate autoimmunity. In recent years, MSCs reportedly exhibited exciting anti-diabetic effects [8–11]. Notably, in a prospective, randomized, single-blinded, placebo-controlled clinical study, MSC infusion not only reduced the exogenous insulin requirement, but also improved the insulin sensitivity index in patients with long-standing T2D [12]. Animal study also demonstrated that MSC transplantation could alleviate hyperglycemia in T2D rats by ameliorating systemic and peripheral insulin resistance [13]. Insulin resistance has been considered to be a chronic low-grade inflammation associated with adipose tissue macrophages (ATMs). ATMs in an M1 pro-inflammatory state, which mostly exist in an obesity state, generate reactive oxygen species and release inflammatory cytokines such as TNF-α or IL-6, leading to adipose tissue inflammation and contributing to the development of insulin resistance [14–16]. In contrast, macrophages in an M2 anti-inflammatory state reportedly play a beneficial role in maintaining insulin sensitivity and glucose tolerance [17–19]. Excitingly, in our previous studies, MSC treatment showed beneficial effects on improving insulin sensitivity by reprogramming M1 macrophages in adipose tissue into M2 state, representing a promising therapy for T2D with a novel therapeutic mechanism [20]. However, increasing evidence has indicated that the hypoglycemic effect caused by single MSC infusion could only last for 1–2 weeks; afterwards, blood glucose gradually increases and reaches a level comparable to that of untreated T2D rodents [13, 21]. The similar limitation was observed in clinical trials [22, 23]. Therefore, developing new strategies to assist MSCs and enhance their efficiency in regulating metabolic disorders in T2D is essential. Given that the macrophage phenotype plays a key role in the pathogenesis and treatment of T2D, promoting macrophages towards the M2 state to enhance the hypoglycemic effects of MSCs may be a possible approach.

Decitabine, a FDA-approved DNA methyltransferase (DNMT) inhibitor, is widely used for anti-tumor therapy in patients with hematological malignancies [24], since it can inhibit tumor cells at high dose by removing the aberrant DNA hypermethylation and reactivation of silenced tumor suppressor genes [25, 26]. However, some studies found that low-dose decitabine or other DNMT inhibitors can induce long-lasting anti-tumor effects without cytotoxicity or changes in cell cycle [27–29]. Subsequent clinical trials reported that the anti-tumor effects of low-dose DNMT inhibitors were largely mediated by inducing a growth-inhibiting immune response independent of DNA methylation levels [30, 31]. Furthermore, studies with low-dose DNMT inhibitors found that they have powerful immunomodulatory effects to treat graft-versus-host disease by suppressing excessive inflammation [32]. More interestingly, DNMT inhibitors also exerted anti-inflammatory effects in acute lung injury, acute myocardial infarction, and atherosclerosis models by promoting M2 polarization in macrophages [33–35]. Based on these reports, we postulated that low-dose decitabine could enhance the effects of MSCs in improving glucose homeostasis by more effectively promoting M2 polarization in T2D mice.

To verify this hypothesis, we investigated the effects of umbilical cord-derived mesenchymal stem cells (UC-MSCs) combined with low-dose decitabine in mice with T2D induced by a high-fat diet (HFD) and streptozotocin (STZ) injection. Low-dose decitabine significantly enhanced the regulation of glucose homeostasis by UC-MSCs by promoting M2 polarization, attenuating adipose tissue inflammation, and improving insulin sensitivity in T2D mice. In addition, the beneficial effects of combined therapy were further confirmed in vitro by cocultivation of UC-MSCs and bone marrow-derived macrophages (BMDMs). Moreover, this process involved the activation of the IL4R-STAT6-PPARγ pathway. Therefore, we suggest that low-dose decitabine can be an effective therapeutic strategy to enhance the effects of MSCs in T2D treatment.

## Methods

### Animals and treatment groups

Eight-week-old male C57BL/6 mice obtained from the Chinese PLA General Hospital were housed for 5 days in a cage with a 12:12-h light/dark cycle at an ambient temperature of 22–25 °C. Then, mice were given a HFD (Research Diets, New Brunswick, NJ, USA, D12492) or normal chow diet (NCD) for 8 weeks. To obtain the T2D model, we intraperitoneally injected 100 mg/kg STZ (Sigma-Aldrich, St. Louis, MO, S0130) into HFD-fed mice. One week after STZ injection, mice that showed fasting glucose levels of ≥ 16.7 mmol/L were considered T2D mice as previously described [36–38]. Intraperitoneal glucose tolerance tests (IPGTTs) and insulin tolerance tests (IPITTs) were performed to confirm the T2D model. For IPGTT or IPITT, mice were fasted overnight and intraperitoneally injected with glucose (1.5 g/kg) or insulin (1 U/kg), and blood glucose was measured every 30 min up to 120 min. T2D mice were divided into the following groups with 18 mice in each group: the T2D with PBS infusion group (T2D group), T2D with MSC infusion group (MSC group), T2D with decitabine administration group (DAC group), and T2D with MSC and decitabine infusion group (MSC + DAC group, experimental control). Mice were administered PBS or UC-MSCs (1 × 10^6^ UC-MSCs in 0.2 mL PBS) once via the tail vein or decitabine (0.25 mg/kg of body weight in PBS) (Xi’an Janssen, China, H20181217) via intraperitoneal injection every day for 5 days. In addition, standard chow diet mice were used as the normal group. One week, 2 weeks, and 4 weeks later, IPGTTs and IPITTs were performed again to assess the effects of UC-MSCs and decitabine. The mice were sacrificed at indicated time points. Six mice per group were examined for each time point. All protocols were approved by the medical ethics committee of the Chinese PLA General Hospital.

### Cell culture

UC-MSCs were isolated from human umbilical cords that were freshly collected from patients who delivered babies in the Chinese PLA General Hospital. Consents were signed by all the patients, and ethical approval was obtained from the Ethics Committee of the Chinese PLA General Hospital. UC-MSCs were processed, purified, and confirmed as previously described [39, 40]. Briefly, after removing the adventitia and vascular fraction, the Wharton rubbers from human umbilical cords were minced into 1 mm × 1 mm pieces and then cultured in medium under 5% CO_2_ at 37 °C. Once reached sub confluence, supernatant was removed and cells adhering to the dishes were digested and subjected to passage.

BMDMs were isolated from the bones of 6-week-old C57BL/6 mice. Femurs and tibias were freshly collected, and BMDMs were produced by flushing the bone marrow from the bones with RMPI-1640 medium. After centrifuge at 1000 rpm/min for 5 min, the pellet was resuspended and cultured in fresh RMPI-1640 medium supplemented with 10% fetal calf serum (Gibco, Grand Island, 10099-044), 1% penicillin-streptomycin (Gibco, PS2004HY), and 100 ng/ml M-CSF (R&D Systems, MN, USA, 416ML-500) at 37 °C in 5% CO_2_. Culture medium was replaced by fresh one every 3 days. On the seventh day, lipopolysaccharide (LPS, 100 ng/mL, Sigma-Aldrich, St. Louis, MO, L4516) and interferon-γ (IFN-γ, 50 ng/mL, Prime Gene Bio-Tech, Shanghai, China, 126-06) were added to stimulate the macrophages. Twenty-four hours later, the stimulated macrophages were treated by decitabine (10 nmol/L) alone or decitabine plus UC-MSCs (5 × 10^4^) in a transwell co-culture system for 48 h. Flow cytometry was used to identify the BMDMs by staining with anti-F4/80 (eBioscience, San Diego, CA, USA, Clone: BM8, 12-4801-82).

### Histopathology and immunofluorescence staining

Epididymal fat was harvested at indicated time points (six mice per group for each time point). The epididymal adipose tissues were fixed in formalin and embedded with paraffin. Sections were cut at 6 μm and stained with hematoxylin-eosin (HE) according to a standard protocol. The size of adipocytes was measured using digital image analysis. For immunofluorescence, the adipose tissues were fixed in 4% paraformaldehyde, dehydrated with 50% sucrose, and embedded in OCT (Sakura, Finetek, USA, 4583). Frozen sections (10 μm thick) (3 slides per mouse) were blocked in BSA and incubated overnight at 4 °C with primary antibodies against Fizz1 (Abcam, San Francisco, USA, ab39626) or iNOS (Abcam, ab15323), followed by incubation with Alexa Fluor 488/594-conjugated secondary antibodies (Invitrogen, A21203, A31556, A-32766) at room temperature for 2 h. Finally, 4,6-diamidino-2-phenylindole (DAPI) (Sigma-Aldrich, St. Louis, MO, B2261) was added. Sections were observed using a confocal laser scanning microscope (Olympus FluoView 1000, Tokyo, Japan, TCSSP8). BMDMs were plated onto glass coverslips and fixed with 4% paraformaldehyde. The next steps were performed as described above.

### Quantitative real-time RT-PCR

Total RNA was extracted from adipose tissues and isolated cells using TRIzol reagent (Thermo Fisher, USA, 15596026) and reverse transcribed into cDNA with a reverse transcription kit (Thermo, Scientific, CA, USA, #K1622) according to the manufacturer’s instructions. Real-time PCR analysis was performed using Power SYBR Green RT-PCR Reagent (Applied Biosystems, Carlsbad, CA, 83700) on an ABI Prism thermal cycler model Step One Plus (Applied Biosystems, CA, USA, ABI 7500). The thermal cycling program was 95 °C for 30 s, followed by 62 °C for 30 s and 72 °C for 30 s for 40 cycles. Melting curve analysis was performed to ensure the specificity of the primers. β-actin was used as a reference gene in each reaction. The PCR primers are listed in the supplement.

### Western blotting

Total protein was extracted from samples of adipose tissue and isolated cells using a PRO-PREP Protein Extraction Kit (iNtRON Biotechnology, Kyungki-Do, Korea, 01434/40116). Aliquots containing 50 mg protein were separated by 8% SDS-PAGE. The primary antibodies were total or phosphorylated AKT (Ser473) (p-AKT), iNOS, Arg-1, PPARγ, total or phosphorylated STAT6 (Tyr641) (p-STAT6), and total or phosphorylated STAT3 (Tyr705) (p-STAT3). Total or phosphorylated AKT (Ser473) (p-AKT) (4685s and 4060s), total or phosphorylated STAT6 (Tyr641) (p-STAT6) (ab32520 and ab28829), and total or phosphorylated STAT3 (Tyr705) (p-STAT3) antibodies (12640S and 9145S) were from Cell Signaling Technology; iNOS (ab 15323) and Arg-1 (Ab60176) antibodies were from Abcam; and PPARγ antibodies (sc-7273) were from Santa Cruz Biotechnology. Goat anti-rabbit (ZB-2301), goat anti-mice (ZB-2305), and rabbit anti-goat IgG (ZB-2306) secondary antibodies were used. Blots were analyzed using ImageJ software (NIH, MD, USA).

### Morphology analysis and quantification

A total of 10 consecutive 10-μm sections were cut through each epididymal white adipose tissue with an interval of 50 μm. The sections were then subjected to immunofluorescent staining. Pictures were taken by fluorescent microscopy, and the number of cells in each cell type was manually counted. Samples from 6 mice were included.

### Statistical analyses

All experiments were repeated at least three times. All data are presented as the means ± SD. The statistical analyses were performed using SPSS v.14.0.1 software for Windows. Differences between means were assessed using one-way analysis of variance (ANOVA). Group differences at the level of *P* < 0.05 were considered statistically significant.

## Results

### Anti-diabetic effect of UC-MSCs was enhanced by low-dose decitabine

**Fig. 1:**
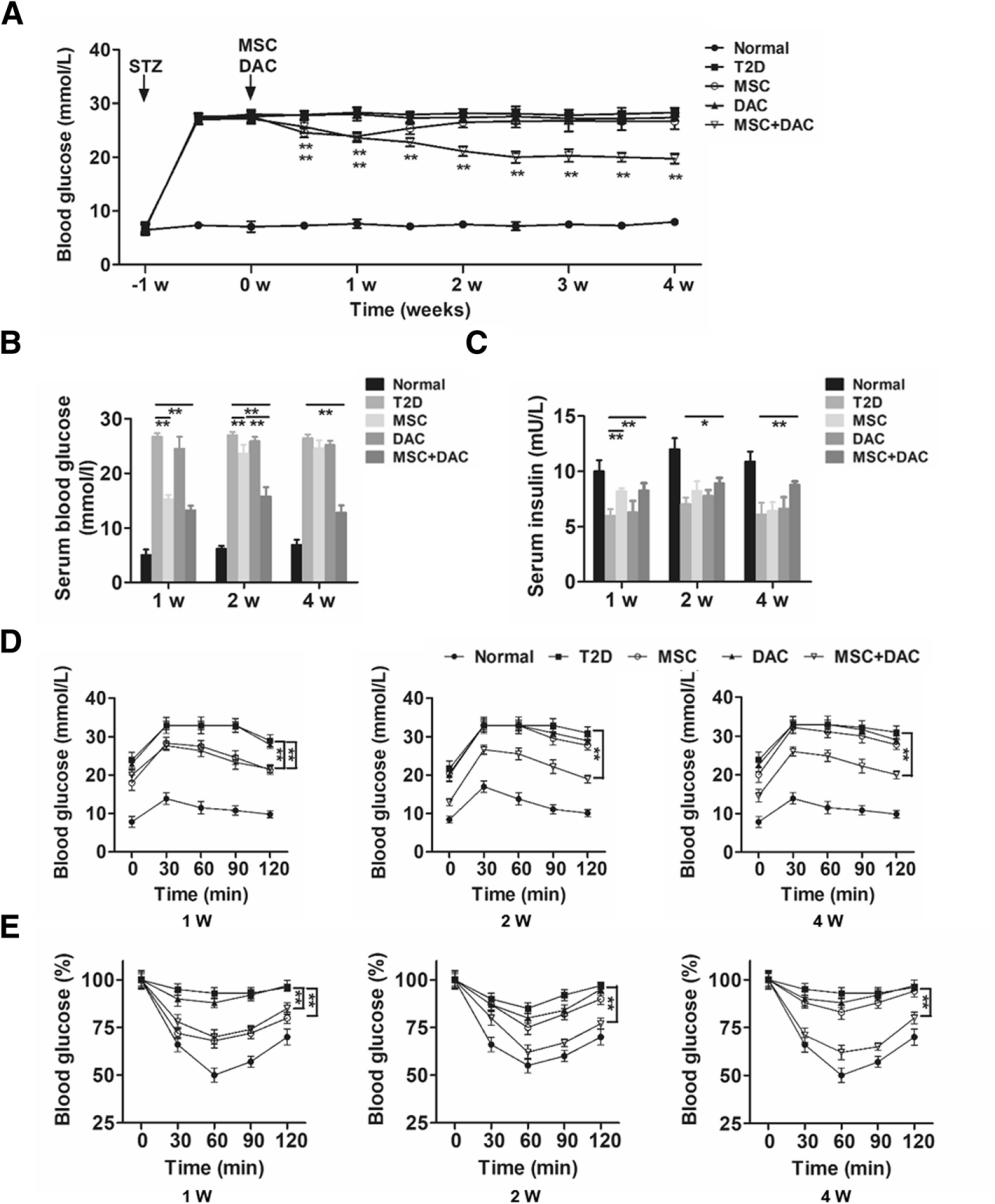
Decitabine enhances and prolongs the anti-diabetic effects of UC-MSCs in T2D mice. T2D mice were randomly divided into the following groups: the T2D group (received PBS infusion), MSC group (received UC-MSC infusion), DAC group (received decitabine administration), and MSC + DAC group (received UC-MSC and decitabine infusion). Mice fed with a NCD served as the control (the normal group). **a** Blood glucose levels were detected consecutively after STZ injection. At 1 week, 2 weeks, and 4 weeks after treatment, the serum blood glucose (**b**) and serum insulin (**c**) concentrations of each group were measured. **d** Glucose tolerance was assessed by IPGTT. **e** Insulin tolerance was evaluated by IPITT. For IPITT, the results are presented relative to the initial blood glucose concentration. Values of **a**–**e** are the means ± SD; *n* = 6 mice per group; *P < 0.05, **P < 0.01. STZ, streptozotocin; T2D, type 2 diabetes; MSC, mesenchymal stem cells; DAC, decitabine; M+D, mesenchymal stem cells combined with decitabine; IPGTT, intraperitoneal glucose tolerance test; IPITT, intraperitoneal insulin tolerance test.

The effect of combination therapy with low-dose decitabine and UC-MSCs on the improvement in glucose homeostasis was tested in a T2D mouse model. The model was induced by a HFD combined with a low dose of STZ as previously reported [38] and confirmed by elevated blood glucose of more than 16.7 mmol/L (Additional file 1: Figure S1A). Deterioration in glucose metabolism and reduced insulin sensitivity were assessed by IPGTT (Additional file 1: Figure S1B) and IPITT, respectively (Additional file 1: Figure S1C). UC-MSCs were isolated from human umbilical cords, and their characteristics were identified by phenotypes and the potential to differentiate into adipocytes and osteoblasts (Additional file 1: Figure S2). Established T2D mice were randomly divided into the following groups: the T2D group (received PBS infusion), MSC group (received UC-MSC infusion), DAC group (received decitabine administration), and MSC + DAC group (received UC-MSCs and decitabine infusion). Standard chow diet-fed mice served as normal controls. After treatment, we monitored the blood glucose in each group for 4 weeks. Mice in the T2D group continued to be severely hyperglycemic (27.9 ± 0.4 mmol/L) throughout the experiment. In the MSC group, the blood glucose began to decline immediately after UC-MSC infusion and dropped to the lowest point (23.8 ± 0.8 mmol/L) at 1 week. However, the blood glucose gradually recovered and returned to the pretreatment level at 2 weeks. Interestingly, the hypoglycemic effect of the combination therapy was comparable to that of the MSC group at 1 week, but instead of a gradual increase, the blood glucose level continued to decrease over the next 3 weeks until the end of the observation (19.7 ± 0.94 mmol/L) (Fig. 1a). Nevertheless, no decline in blood glucose was observed in the single decitabine treatment group. Accordingly, fasting serum blood glucose and serum insulin concentrations (Fig. 1b, c) were examined at 1 week, 2 weeks, and 4 weeks after treatment. Consistent with the above results, the increase in the serum insulin concentration by UC-MSCs was observed at 1 week only and could not be detected at 2 weeks, while the improvement in islet secretion function in the MSC + DAC group remained significant at 4 weeks compared to that in the T2D group (Fig. 1c). Moreover, the results of IPGTTs and IPITTs showed that after the addition of decitabine, the improvement in glucose tolerance and insulin sensitivity by UC-MSCs was extended from 1 to 4 weeks (Fig. 1d, e). Together, these results demonstrated that low-dose decitabine could prolong and enhance the effect of UC-MSCs in improving glucose homeostasis in T2D mice.

### Insulin sensitivity was improved by a combination of UC-MSCs and low-dose decitabine

**Fig. 2:**
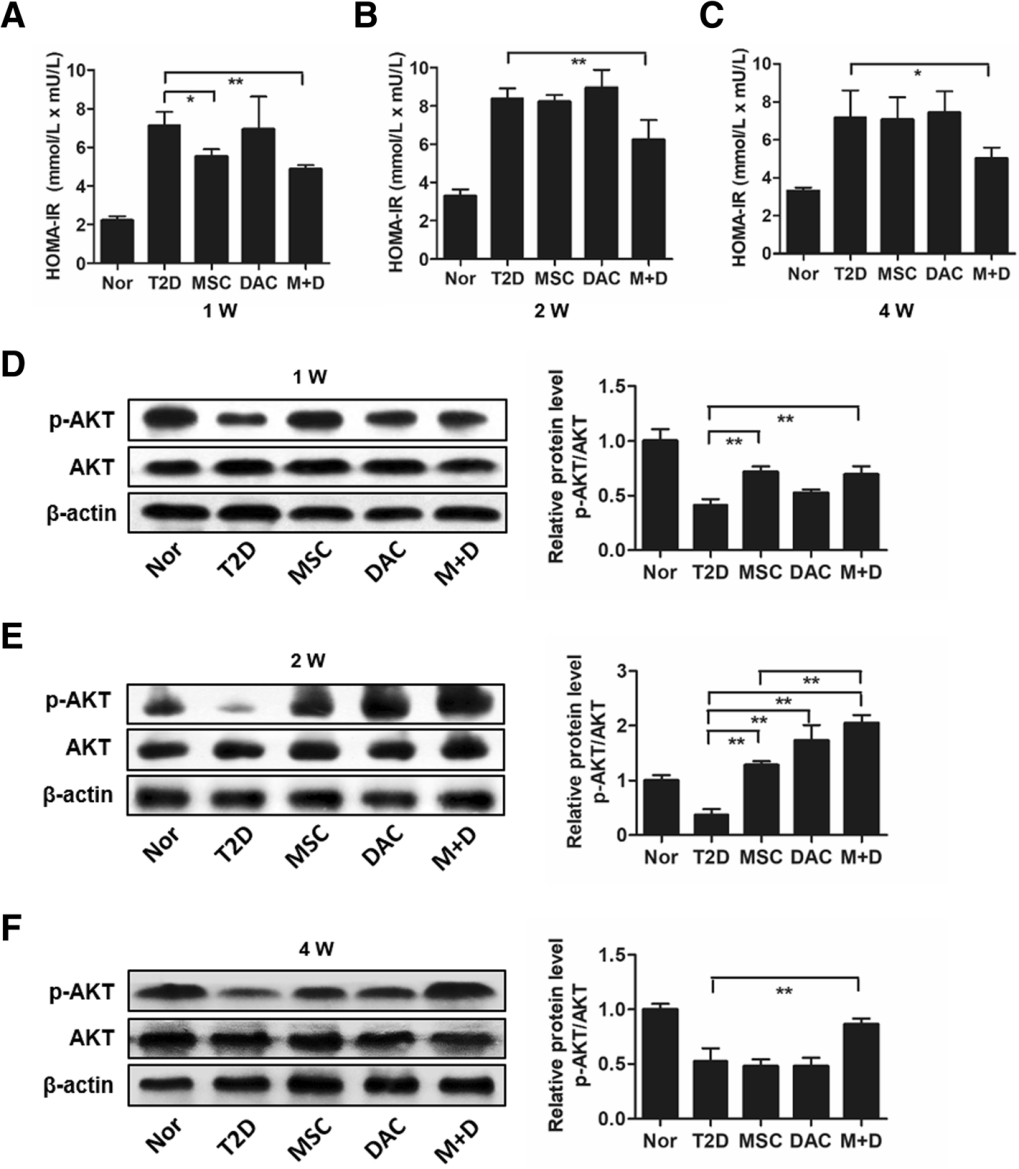
Decitabine enhances the effects of UC-MSCs on improved insulin resistance. **a**–**c** HOMA-IR assessment of mice in each group at 1 week (**a**), 2 weeks (**b**), and 4 weeks (**c**) after treatment for systematic insulin resistance. HOMA-IR index = (FBG [in mmol/L] × FINS [in units/L])/22.5. **d**–**f** Immunoblotting analysis of p-AKT and total AKT expression in epididymal adipose tissue at 1 week (**d**), 2 weeks (**e**), and 4 weeks (**f**) after treatment. The ratios of p-AKT to total AKT were quantitated. Values are the means ± SD; *n* = 6 mice per group; *P < 0.05, **P < 0.01. p-AKT, phosphorylated AKT

Previous studies have shown that MSCs treat T2D mainly by improving insulin sensitivity [13]. Thus, we wanted to investigate whether UC-MSCs plus decitabine further enhanced this effect. First, we examined the homeostasis model assessment of the insulin resistance (HOMA-IR) index for each group. At 1 week after treatment, compared with the T2D group, both the UC-MSC alone and UC-MSC plus decitabine groups showed a significant reduction in the HOMA-IR index (*P* < 0.01), and there was no significant difference between the two groups (Fig. 2a). Interestingly, compared to the MSC group, the MSC + DAC group exhibited a lower HOMA-IR index at 2 weeks (Fig. 2b), and this positive effect remained at the end of the study (4 weeks) (Fig. 2c). However, the effect of UC-MSCs completely disappeared at 2 weeks (Fig. 2b), indicating that systemic insulin sensitivity was further improved by UC-MSCs combined with decitabine. Second, we detected insulin signaling by examining the expression of p-AKT and AKT in adipose tissues, which are the most critical tissue in the initiation and development of insulin resistance. Western blot analysis showed that similar to the results of the HOMA-IR index, at 1 week after treatment, compared with T2D, both UC-MSCs alone and UC-MSCs plus decitabine significantly increased p-AKT expression (*P* < 0.05), and there was no significant difference between the two groups (Fig. 2d). However, at 2 weeks, combination therapy induced 181.5% higher expression of p-AKT (*P* = 0.001) than did UC-MSCs alone (Fig. 2e). Moreover, UC-MSCs combined with decitabine treatment but not UC-MSCs alone remained effective at 4 weeks (Fig. 2f). Additionally, decitabine monotherapy improved insulin sensitivity at certain time points, supporting our hypothesis that decitabine may have an effect on insulin resistance via regulation of macrophage polarization. These results confirmed that low-dose decitabine could effectively enhance the effects of UC-MSCs in improving insulin sensitivity in T2D mice.

### Adipose tissue inflammation was further attenuated by combination treatment with decitabine

**Fig. 3:**
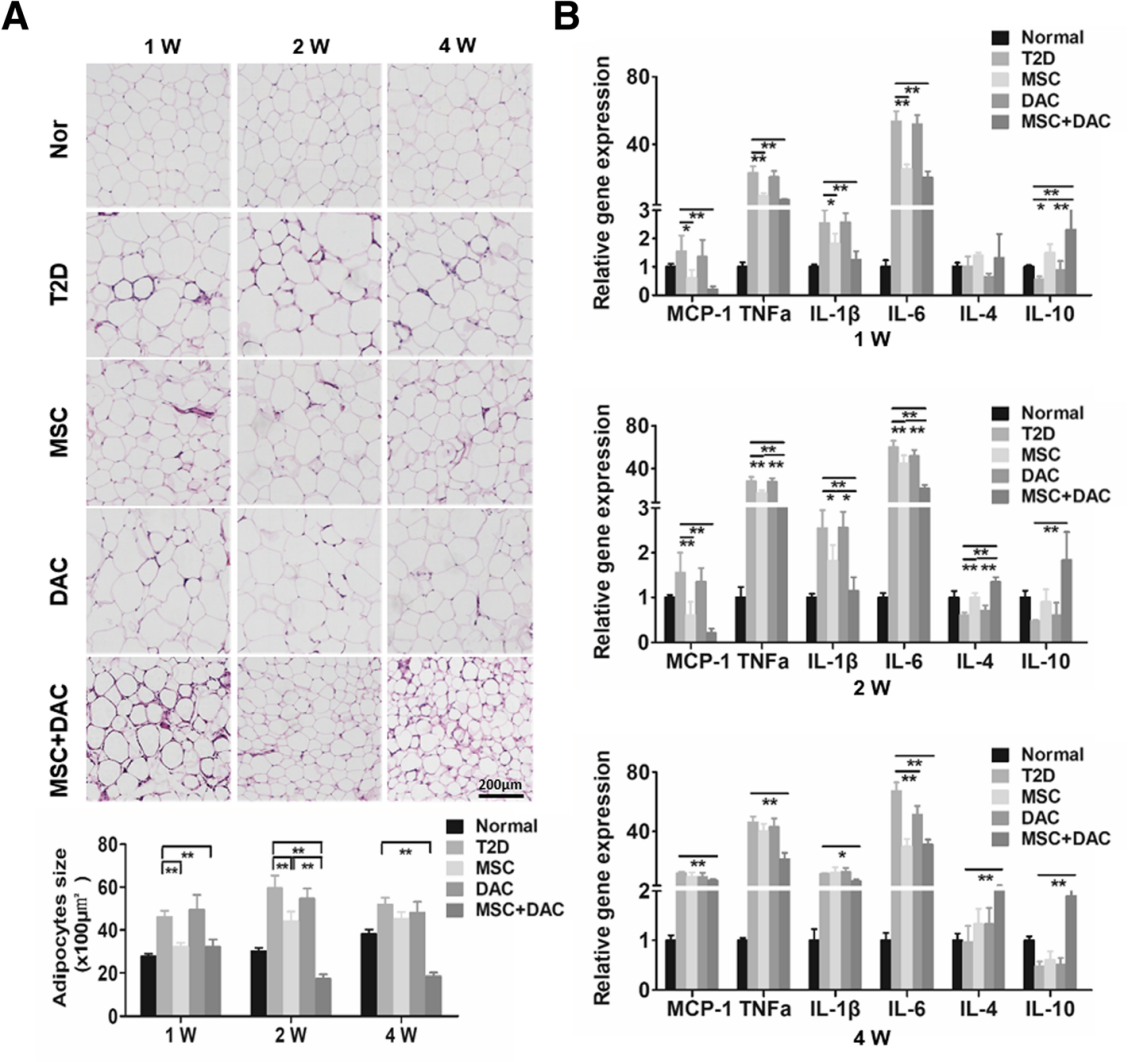
UC-MSCs combined with decitabine more effectively ameliorated inflammation in adipose tissue. **a** Representative HE staining of adipose tissue from the normal, T2D, MSC, DAC, and MSC + DAC groups. Scale bar = 200 μm. The size of adipocytes was measured using digital image analysis. **b** Quantitative reverse transcriptase polymerase chain reaction analysis of inflammation-related gene expression in adipose tissue from five groups; the results are presented relative to those of T2D mice. Values are the mean + SD; *n* = 6 mice per group, *P < 0.05; **P < 0.01

Given the close association between adipose tissue inflammation and insulin resistance, we subsequently examined the inflammatory state of adipose tissue. First, we examined the pathology of epididymal adipose tissue. HE staining showed obviously enlarged adipocytes in diabetic mice. After UC-MSC infusion, the average size of adipocytes significantly decreased at 1 week and remained smaller than that of the T2D group at 2 weeks. However, at 4 weeks, there was no difference between the MSC and T2D groups. Notably, treatment with MSC + DAC exerted a more pronounced and durable size reduction effect, and the average adipocyte size was reduced to 35.4% of that in T2D mice by the fourth week (Fig. 3a). Furthermore, we examined the gene expression of inflammation-related molecules in epididymal adipose tissue (Fig. 3b). As shown in Fig. 3b, the expression of pro-inflammatory genes, such as MCP1, TNFα, IL-1β, and IL-6, was increased in T2D mice. UC-MSC infusion inhibited the expression of the above pro-inflammatory cytokines within 2 weeks, but this inhibitory effect disappeared at 4 weeks after treatment. However, UC-MSCs combined with decitabine resulted in an overall decrease in the above pro-inflammatory genes throughout the experimental course. Moreover, the expression of the anti-inflammatory genes IL-4 and IL-10 was more elevated in the MSC + DAC group than in the MSC group (Fig. 3b). These data indicated that combination therapy with UC-MSCs and decitabine could more effectively attenuate adipose tissue inflammation.

### UC-MSCs combined with low-dose decitabine upregulate M2 macrophages in adipose tissue

**Fig. 4:**
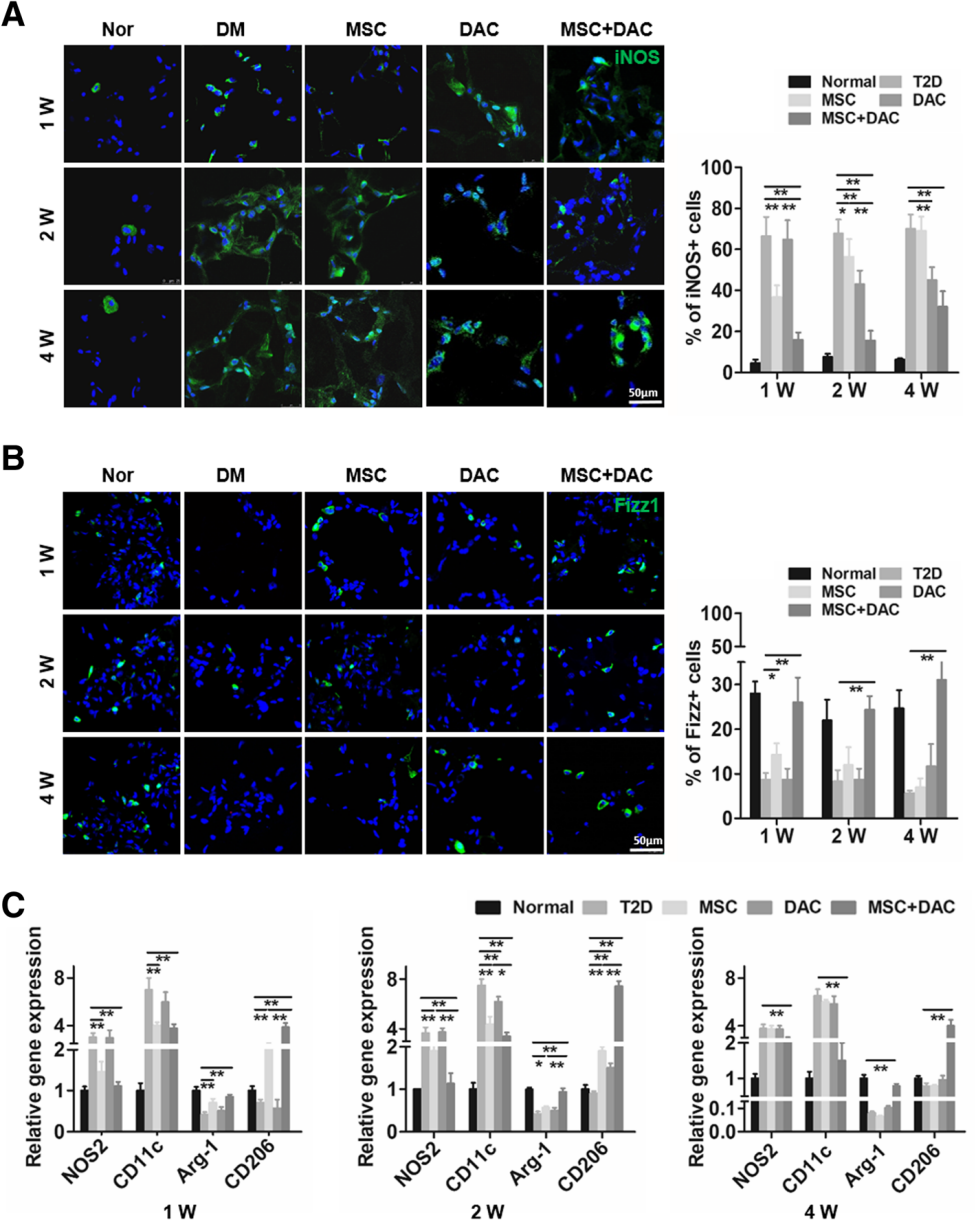
UC-MSCs combined with decitabine induced more M2 macrophages in adipose tissue. **a** Representative of iNOS-positive cells (M1 macrophage maker) in adipose tissue by immunofluorescence and quantification of iNOS-positive cells. Scale bar = 50 μm. **b** Representative images of Fizz1-positive cells (M2 macrophage maker) in adipose tissue by immunofluorescence and quantification of Fizz1-positive cells. Scale bar = 50 μm. The percentage of iNOS-positive cells (**a**) and Fizz1-positive cells (**b**). **a** and **b** were determined by evaluating cells manually from at least five sections of the each slide, at least 3 slides per mouse, at least 6 mice per group. Results were presented as the means ± SD. **c** Quantitative reverse transcriptase polymerase chain reaction analysis of macrophage phenotype-related gene expression in adipose tissue from the normal, T2D, MSC, DAC, and MSC + DAC groups. The results are presented relative to those of T2D mice. Values are mean ± SD; *n* = 6 mice per group, *P < 0.05; **P < 0.01

Next, we examined the effects of UC-MSCs combined with decitabine on macrophages in adipose tissue, which has remained our primary focus. Immunofluorescent staining showed a large increase in iNOS-positive cells (M1 macrophage maker) and fewer Fizz1-positive cells (M2 macrophage maker) in T2D mice than in normal control mice, consistent with previous studies [15, 16]. In the MSC group, the expression of iNOS significantly decreased at 1 week and remained at a lower level at 2 weeks, although the difference between the MSC and T2D groups was reduced. At the fourth week after UC-MSC administration, this downregulation effect disappeared completely. Remarkably, UC-MSCs combined with decitabine resulted in fewer iNOS-positive cells than did UC-MSC treatment at 1 week, 2 weeks, or 4 weeks (Fig. 4a). Accordingly, we examined the effect of combination therapy on M2 macrophages in epididymal fat. Immunofluorescence showed that the increase in Fizz1-positive cells in the MSC group was observed at 1 week only compared with that in the T2D group. In contrast, combination treatment markedly increased Fizz1 expression in adipose tissue at 1 week, 2 weeks, or 4 weeks after treatment (Fig. 4b). Additionally, in the DAC group, the expression of iNOS was partially downregulated, but the expression of Fizz1 was not significantly changed compared to those in the T2D group (Fig. 4a, b). Furthermore, more macrophage markers were detected by RT-PCR analysis. The results revealed that the treatment of UC-MSCs combined with decitabine induced much lower expression of M1 macrophage-related genes (NOS2 and CD11c), as well as higher expression of M2 macrophage markers (Arg-1 and CD206) over the entire study period (Fig. 4c). Taken together, these findings indicated that combined treatment further reduced M1 macrophages in adipose tissue, while the number of anti-inflammatory M2 macrophages significantly increased, accompanied by an improvement in insulin resistance and further alleviation of the associated inflammation.

### Low-dose decitabine combined with UC-MSCs more effectively polarized M1 macrophages towards M2 macrophages in vitro

**Fig. 5:**
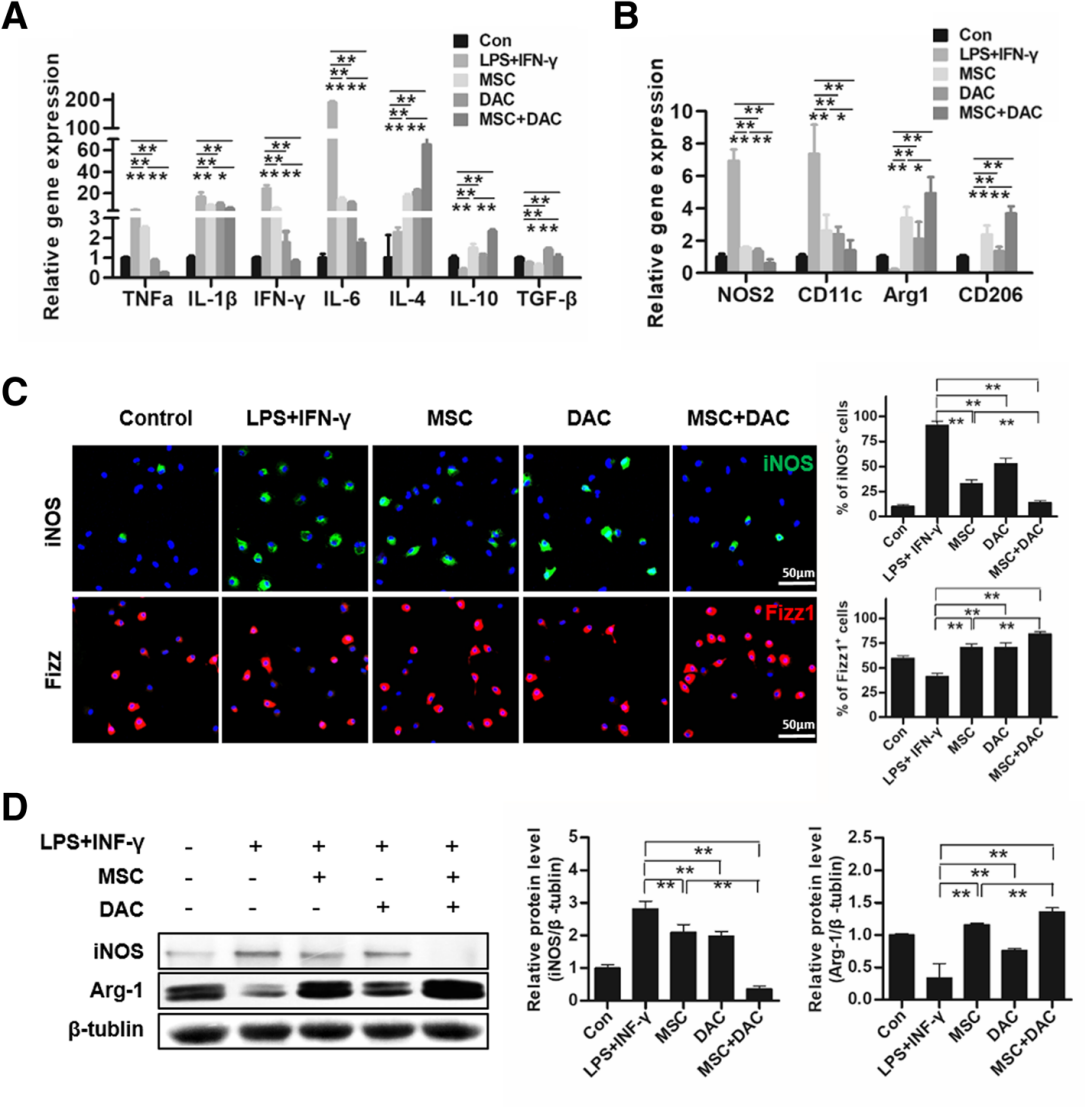
UC-MSCs combined with decitabine more effectively polarized M1 macrophages towards M2 macrophages in vitro. BMDMs were isolated from C57BL/6 and stimulated by LPS and IFN-γ for 12 h (LPS + IFN-γ group). Then, LPS + IFN-γ-induced BMDMs (M1 macrophages) were cultured with 5 × 10^4^ UC-MSCs in a Transwell system for 24 h (MSC group), with decitabine (10 nmol/L) for 48 h (DAC group), or with decitabine for 48 h after coculture with UC-MSCs for 24 h (M+D group). **a** Quantitative RT-PCR analysis of inflammation-related gene expression in macrophages from five groups. **b** Representative macrophage phenotype-related gene expression in macrophages by QT-PCR analysis. **c** Immunofluorescence and quantification of iNOS-positive and Fizz1-positive cells in five groups. Scale bar = 50 μm. Quantification was determined by evaluating cells from at least 5 sections of each slide, at least 3 slides per group. **d** Immunoblotting analysis of iNOS and Arg-1 expression in macrophages. Relative protein levels are quantified by the ratio of iNOS or Arg-1 to β-tubulin. The results are presented relative to those of the LPS + IFN-γ group. Images of **a**–**d** are representatives of three independent experiments. Values are the mean ± SD (*n* = 3) of three individual experiments, *P < 0.05; **P < 0.01

To investigate whether UC-MSCs combined with decitabine directly modulate the macrophage phenotype, we performed an in vitro study using BMDMs from C57BL/6 J mice. First, BMDMs were stimulated with LPS + IFN-γ for 12 h, which promotes maximal expression of pro-inflammatory M1 macrophages [38]. Next, LPS + IFN-γ-induced BMDMs (M1 macrophages) were treated with UC-MSCs, decitabine, or UC-MSCs + decitabine. As expected, incubation of M1 macrophages with UC-MSCs or decitabine alone resulted in inhibition of the secretion of pro-inflammatory molecules, such as TNF-α, IL-1β, IFN-γ, and IL-6. More importantly, this inhibitory effect was significantly enhanced when M1 macrophages were treated with decitabine after coculture with UC-MSCs (Fig. 5a). In addition, the expression of anti-inflammatory cytokines (IL-4, IL-10, and TGF-β) showed more improvement in the MSC + DAC group than in the MSC group (Fig. 5a). Based on these remarkable anti-inflammatory effects of UC-MSCs combined with decitabine, we next analyzed the changes in macrophage markers after MSC + DAC treatment. BMDMs treated by MSC + DAC showed lower expression of NOS2 and CD11c, as well as higher expression of Arg-1 and CD206 compared with those in the MSC group (Fig. 5b). Immunofluorescence results demonstrated that iNOS-positive cells were reduced by 61.3%, 37.9%, and 83.4% in the MSC-treated, decitabine-treated, and MSC + DAC-treated groups, respectively, compared to those in the LPS + INF-γ group. Additionally, Fizz1-positive cells were increased by 71.0%, 70.8%, and 104.1%, respectively (Fig. 5c). Consistently, immunoblotting analysis revealed that MSC + DAC administration induced a more profound decrease in iNOS expression by 61.4% compared to that induced by MSC administration (Fig. 5d), along with a more marked increase in Arg-1 expression by 56.8%. Taken together, these results confirmed that UC-MSCs combined with decitabine could more effectively polarize M1 macrophages to M2 macrophages, thus further suppressing pro-inflammatory properties and promoting anti-inflammatory properties in macrophages.

### Low-dose decitabine combined with UC-MSCs promoted M2 macrophage polarization via the IL-4R/STAT6 axis in a PPARγ-dependent manner

**Fig. 6:**
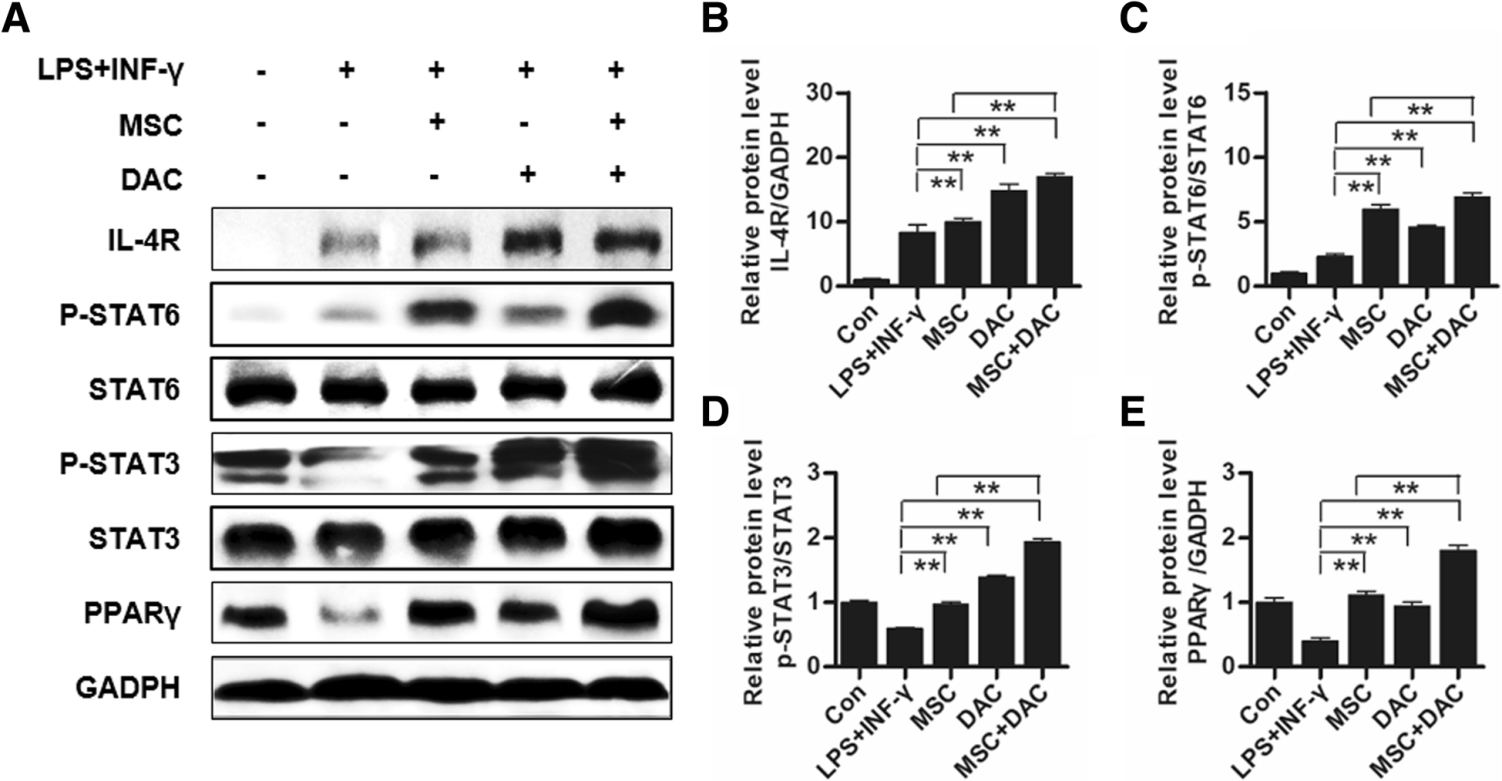
UC-MSCs combined with decitabine induced M2 macrophage polarization and activation of the IL-4R/STAT6/PPARγ pathway. **a** Immunoblotting analysis of macrophages from the Con, LPS + IFN-γ, MSC, DAC, and MSC + DAC groups. **b**–**e** Relative protein levels of IL-4R, p-STAT6, p-STAT3, and PPARγ are shown. Images of **a** are representative of three independent experiments. *P < 0.05; **P < 0.01

Next, we sought to explore the possible mechanism involved in the effect of MSC + DAC on macrophage polarization. The IL-4/STAT6 signaling axis, which controls alternative macrophage activation, is modulated in a PPARγ-dependent manner [19, 41, 42]. Therefore, we tested the expression of the IL-4R/STAT6 axis and PPARγ in macrophages in each group. Immunoblotting results showed that the expression of IL-4R, p-STAT6, p-STAT3, and PPARγ was significantly upregulated in the MSC group compared with that in the LPS + INF-γ group (Fig. 6a). Remarkably, in the MSC + DAC group, IL-4R and p-STAT6 expression levels were further increased (Fig. 6b, c), and p-STAT3 expression was upregulated by 50.74% compared with those in the MSC group (Fig. 6d). Moreover, PPARγ expression was 1.06-fold higher in the combination therapy group than in the MSC group (Fig. 6e), suggesting that the IL-4/STAT6 axis is further activated in a PPARγ-dependent manner by the combined treatment of UC-MSCs and decitabine.

**Fig. 7:**
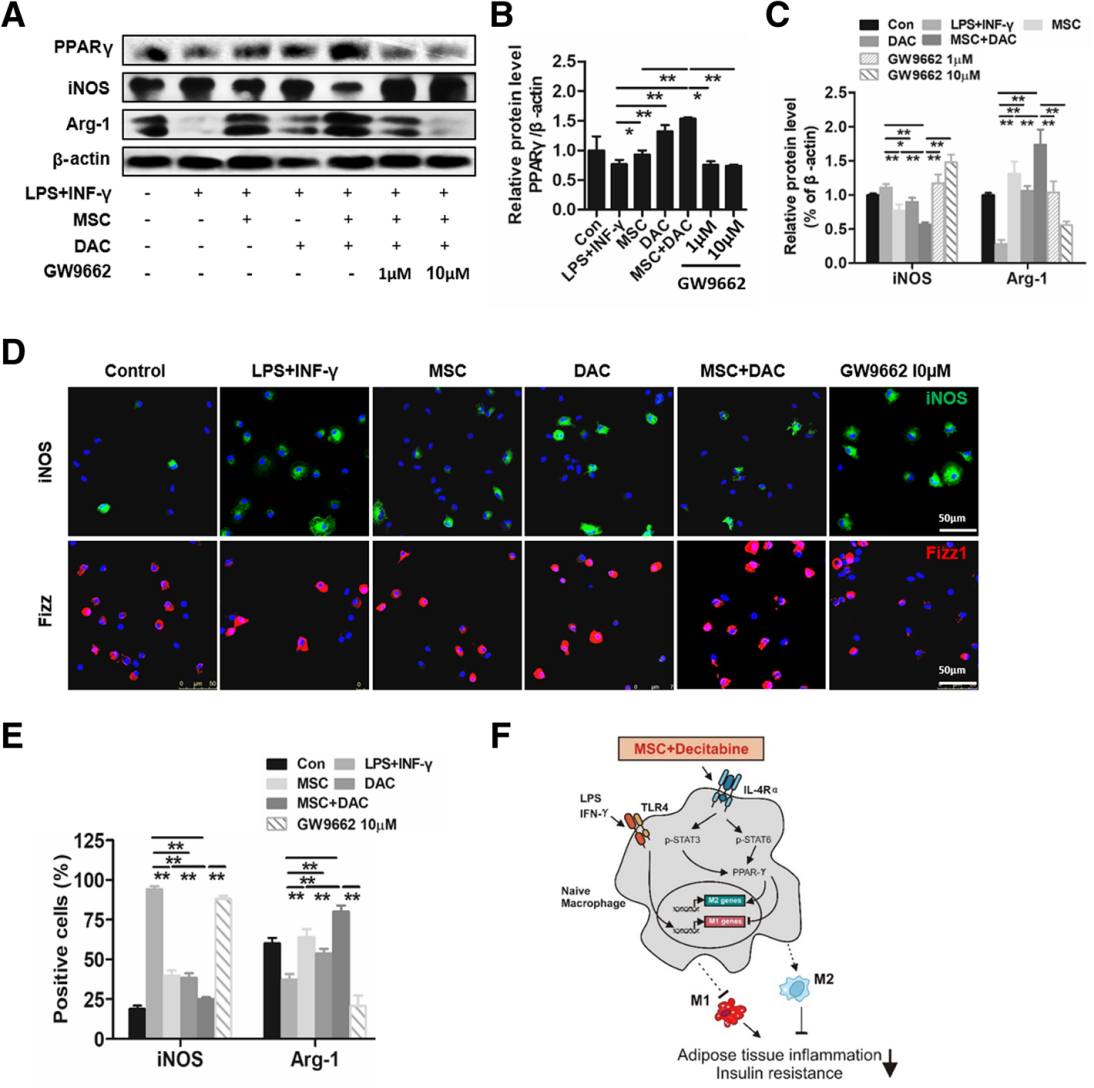
The effect of MSC + DAC on macrophage polarization is dependent on PPARγ activation. BMDMs were cultured alone (con group) or stimulated by LPS and IFN-γ for 12 h (LPS + IFN-γ group). LPS + IFN-γ-induced macrophages were treated with UC-MSCs (MSC group), decitabine (DAC group), UC-MSCs combined with decitabine (M+D group), or GW9662 (1 μmol/L or 10 μmol/L) for 24 h before coculture with M+D (GW9662 1 μM or 10 μM). **a** Immunoblotting analysis of macrophages among seven groups. **b**, **c** Relative protein levels of PPARγ, iNOS, and Arg-1 are shown. **d**, **e** Immunofluorescence and quantification of iNOS and Fizz-1 in macrophages from six groups. Images of **a** and **e** are representative of three independent experiments. *P < 0.05; **P < 0.01. **f** A scheme of IL4R/Stat6 axis controlling alternative (M2) activation. GW9662, PPARγ antagonist

To further clarify the role of PPARγ in the regulation of the macrophage phenotype, we used a PPARγ antagonist, GW9662 (1 μmol/L and 10 μmol/L), to treat BMDMs before UC-MSC or/and decitabine administration. First, we observed that upon GW9662 exposure, the MSC + DAC-mediated activation of PPARγ expression was strongly decreased, especially in the GW9662 10 μM group (Fig. 7a, b). Interestingly, with the downregulation of PPARγ, the expression of iNOS, which was inhibited by MSC + DAC, was significantly increased. Moreover, the increase in Arg-1 was strongly reduced (Fig. 7a, c). A similar effect of the PPARγ antagonist on the regulation of iNOS and Fizz1 expression was also confirmed by immunofluorescence staining (Fig. 7d, e).

Altogether, these results revealed that low-dose decitabine combined with UC-MSCs possibly affected the IL-4/STAT6 signaling axis by increasing PPARγ expression, thereby promoting M2 polarization (Fig. 7f).

## Discussion

According to recent clinical and experimental data, MSCs can effectively improve insulin resistance and ameliorate hyperglycemia, representing a promising therapy for T2D [12, 13, 20]. However, the beneficial response to a single MSC infusion in both diabetic models and patients usually occurs immediately and is maintained for a limited time [21–23]. This relatively temporary role has led many people in the field to explore effective approaches to strengthen or prolong the anti-diabetic effects of MSCs [43, 44]. In the present study, we administered low doses of decitabine combined with UC-MSCs to T2D mice induced by HFD and STZ administration. Based on our results, low doses of decitabine combined with MSCs induced much lower blood glucose than did T2D and MSCs alone, and these lower blood glucose levels lasted much longer than did those induced by a single MSC infusion (at least 4 weeks after treatment). Moreover, compared with MSC-treated mice, MSC + DAC-treated mice showed lower HOMA-IR, paralleling the increase in peripheral insulin sensitivity, and greater improvement in IPGTT and IPITT. Taken together, our findings suggest that decitabine combination therapy could effectively improve insulin sensitivity and enhance the anti-diabetic effects of MSCs in T2D mice. Besides, the dose of decitabine (0.25 mg/kg/day) used in this study is similar to the low dose recommended for clinical use in humans [45], much lower than that used in previous rodent-based studies [46], indicating that the combination of MSCs and decitabine could be safe for T2D.

According to our previous results, MSCs precisely alleviated chronic inflammation in T2D by eliciting M1 macrophage to M2 macrophage phenotype (M2) in adipose tissue [20]. Low-dose DNMT inhibitors had been reported promoting M1 macrophages to M2 polarization and suppressed excessive inflammation in the acute lung injury, acute myocardial infarction, and atherosclerosis model [33–35]. These observations prompted us to investigate whether decitabine combined with MSCs further promotes the conversion of M1 macrophages into M2. First, in vitro, decitabine was effective in promoting the polarization of M1 macrophages to M2 macrophages, in agreement with previous analyses. Remarkably, compared with MSCs alone and decitabine alone, decitabine combined with MSCs strongly upregulated M2 marker expression and inhibited M1 marker expression in LPS + IFN-γ-induced BMDMs. Moreover, these MSC + DAC-primed M2 macrophages exhibited more pronounced anti-inflammatory properties. Second, we examined the impact of MSC + DAC on macrophage phenotype in vivo. Decitabine monotherapy reduced iNOS expression for a period of time but did not influence the expression of Fizz1. However, decitabine combined with MSCs resulted in pronounced inhibition of the expression of the M1 macrophage marker iNOS in adipose tissue, while the expression of Fizz1, an M2 macrophage marker, was further increased in T2D mice, even compared to the MSC group. Moreover, adipose tissue inflammation in the MSC + DAC group was much lower than that in MSC group, suggesting that decitabine plus MSCs can further prime M1 macrophages into M2 macrophages and reduce macrophage-related chronic inflammation in T2D mice.

In this study, after MSC infusion, insulin sensitivity and glucose metabolism in T2D mice were significantly improved within 1 week, and the hypoglycemic effect gradually disappeared, consistent with previous studies [13, 21]. Excitingly, in the MSC + DAC group, impaired glucose tolerance and serum blood glucose levels were much lower than those in the MSC group after 2 weeks, followed by further downregulation of M1 macrophage marker genes and upregulation of M2 macrophage marker genes in adipose tissue. This result is in line with previous analyses suggesting that enhancing M2 macrophages could potentially maintain insulin sensitivity and improve glucose homeostasis [17, 42]. Insulin resistance has been identified as a chronic inflammation primarily mediated by ATMs, which manifested by an increase in M1 macrophages and a decrease in M2 macrophages [14–16]. Macrophage polarization towards M2 macrophages contributes to an improvement in insulin sensitivity [17, 19]. A study with 5-azadC showed that the demethylating agent can suppress ATM inflammation and improve insulin sensitivity in ob/ob mice [47]. Moreover, our previous data revealed that MSC-induced M2 macrophages reversed the insulin resistance caused by M1 macrophages in vitro [20]. Therefore, we have a reason to believe that the further improvement of insulin resistance by combination therapy was attributed to the immunomodulatory effects on macrophage phenotypes. Although treatment with decitabine alone increased the expression of p-AKT in adipose tissue at 2 weeks only without any effect on the HOMA-IR index and did not reduce blood glucose in T2D mice. And this is inconsistent with the previous study in ob/ob mice [47], may be attributed to different drugs and different animal models used in the two studies.

IL-4R-STAT3/STAT6-PPAR-γ axis regulates the polarization of alternatively activated macrophages (M2) and plays a significant role in the resolution of inflammation and maintenance of insulin sensitivity [48, 49]. Odegaard et al. [22] reported that disruption of IL-4R signaling in myeloid cells impaired alternative macrophage activation. As the downstream of IL-4R signaling, STAT6’s KO significantly suppressed the expression of M2 macrophage markers in adipose tissue. Similar with STAT6, STAT3 also regulates M2-like macrophage phenotype as a downstream protein of IL-4R signaling [42, 50]. In addition, myeloid-specific PPAR-γ knockout impairs M2 macrophage polarization and an intact IL-4R/STAT6/PPARγ axis is required for the maturation of alternatively activated adipose tissue macrophages [22]. Based on the reports above, we next investigated whether decitabine combined with MSCs affected the IL-4R-STAT6-PPAR-γ pathway. Our results show that the expression of IL-4R, p-STAT6, p-STAT3, and PPAR-γ was significantly upregulated in the MSC + DAC group compared with that in the LPS + INF-γ and MSC groups. Furthermore, cocultivation of M1 macrophages undergoing M2 differentiation with the PPAR-γ antagonist GW9662 completely abolished the regulation of iNOS and Arg-1 expression by MSC + DAC, indicating that the observed effects of MSC + DAC are PPAR-γ dependent. Nevertheless, the precise mechanisms of the effect of MSC + DAC on PPAR-γ expression remain poorly understood.

In this study, we used decitabine plus MSCs to treat T2D mice, and we found that decitabine plus MSCs downregulated the blood glucose and the low glucose level maintained longer. This remarkable and persistent hypoglycemic effect of MSC + DAC may attribute to their regulation of macrophage polarization. How MSC + DAC regulates macrophage polarization and what happens to MSCs after injection in vivo remain unclear. We investigated the homing of UC-MSCs by injecting CM-Dil-labeled UC-MSCs into HFD + STZ-induced T2DM mice. One week later, whether MSCs alone or MSCs plus decitabine, no CM-Dil-positive cells were found in adipose tissue, and very few CM-Dil-positive cells were found in the lung and liver in either group (Additional file 1: Figure S4**)**, at least indicating that the effect of MSCs on macrophage polarization does not attribute to the direct interaction between MSCs and macrophages, and the enhanced anti-diabetic effect by decitabine does not depend on the homing of MSCs into targeted organ. The precise mechanisms are now under investigation in our group.

## Conclusions

In conclusion, decitabine combined with UC-MSCs attenuated insulin resistance more effectively and enhanced the anti-diabetic effects of MSCs in T2D mice, and this effect was partially attributed to directing macrophages into M2 phenotype via increasing PPARγ expression. This illustrated that decitabine may be an applicable addition to MSCs for diabetes therapy. Our study also suggested that modulation of the macrophage phenotype may be a novel therapeutic target in the treatment of T2D.

## Additional file

Additional file 1 Table S1. Primer sequences of target genes (mice). Figure S1. Induction of the T2D mouse model. The T2D mouse model was induced by a combination of an 8-week HFD and a single intraperitoneal injection of low-dose STZ (100 mg/kg). a Blood glucose was consecutively detected after STZ injection. One week after STZ injection, IPGTT (b) and IPITT (c) were performed to confirm the establishment of the T2D mouse model. For IPITT (c), the results are presented relative to the initial blood glucose levels. The results are presented as the means ± SD. *P < 0.05, **P < 0.01. Figure S2. Identification of UC-MSC characteristics. UC-MSCs were isolated and identified from full-term fetal umbilical cords. a The morphology of UC-MSCs. (B-C): Multilineage differentiation of MSCs: adipocytic differentiation (b) was detected by Oil Red O staining; osteoblastic differentiation (c) was confirmed by Alizarin Red staining; Scale bar 50 μm. (d–f): Immunological phenotypes of MSCs: cultured UC-MSCs consisted of a homogenous mesenchymal population that stained positive for CD90 in 99.76% of cells (d) and CD105 in 99.79% of cells (e) but negative for CD45 (f), which met the international definition of MSCs. Figure S3. Biological characteristics and identification of bone marrow-derived macrophages (BMDMs). BMDMs were isolated and cultured in RMPI-1640 supplemented with 100 ng/ml M-CSF. a The morphology of macrophages. b Flow cytometry analyses showed that the positive rate of F4/80 in bone marrow-derived cells was over 95%. (c, d): M1 macrophage induction, after stimulation with LPS (100 ng/ml) and IFN-γ (50 ng/ml) for 24 h, and the morphology of macrophages (c). The positive rate of CD11c in BMMCs was over 95% (d). Abbreviations: BMDMs, bone marrow-derived macrophages. M-CSF, macrophage colony-stimulating factor. Figure S4. UC-MSCs combined with decitabine did not influence UC-MSCs homing in T2DM mice. UC-MSCs were CM-Dil (red) labeled in advance. Successfully induced type 2 diabetic mice were treated with UC-MSCs or UC-MSCs combined with decitabine. One week after the infusion, detection of UC-MSCs in the adipose tissue, liver, and lung of T2DM recipients using a confocal laser scanning microscope. Scale bar, 100 μm (A) and 50 μm (B). Values are presented as the means ± SD. ns, no significant difference. (DOCX 2295 kb)

## Data Availability

The datasets used and/or analyzed during the current study are available.

## Abbreviations

ATMs: Adipose tissue macrophages
BMDMs: Bone marrow-derived macrophages
DAC: Decitabine
DNMTs: DNA methyltransferases
HFD: High-fat diet
HOMA-IR: Homeostasis model assessment of the insulin resistance
IFN-γ: Interferon-γ
IPGTT: Intraperitoneal glucose tolerance tests
IPITT: Intraperitoneal insulin tolerance test
LPS: Lipopolysaccharide
M+D: UC-MSCs combined with decitabine
MSCs: Mesenchymal stem cells
PBS: Phosphate-buffered saline
PPARγ: Peroxisome proliferator-activated receptor gamma
STZ: Streptozotocin
T2D: Type 2 diabetes
TZD: Thiazolidinedione
UC-MSCs: Umbilical cord-derived mesenchymal stem cells

## References

[1] Cho NH, Shaw JE, Karuranga S, Huang Y, Da RFJ, Ohlrogge AW (2018). IDF Diabetes Atlas: global estimates of diabetes prevalence for 2017 and projections for 2045. Diabetes Res Clin Pract.

[2] Selvin E, Parrinello CM, Sacks DB, Coresh J (2014). Trends in prevalence and control of diabetes in the United States, 1988-1994 and 1999-2010. Ann Intern Med.

[3] Ji LN, Lu JM, Guo XH, Yang WY, Weng JP, Jia WP (2013). Glycemic control among patients in China with type 2 diabetes mellitus receiving oral drugs or injectables. BMC Public Health.

[4] Reaven GM (1993). Role of insulin resistance in human disease (syndrome X): an expanded definition. Annu Rev Med.

[5] Chatterjee S, Davies MJ (2015). Current management of diabetes mellitus and future directions in care. Postgrad Med J.

[6] Lewis JD, Ferrara A, Peng T, Hedderson M, Bilker WB, Jr CPQ (2011). Risk of bladder cancer among diabetic patients treated with pioglitazone: interim report of a longitudinal cohort study. Diabetes Care.

[7] Sridhar S, Walia R, Sachdeva N, Bhansali A (2013). Effect of pioglitazone on testosterone in eugonadal men with type 2 diabetes mellitus: a randomized double-blind placebo-controlled study. Clin Endocrinol.

[8] Bhansali A, Upreti V, Walia R, Gupta V, Bhansali S, Sharma RR (2014). Efficacy and safety of autologous bone marrow derived hematopoietic stem cell transplantation in patients with type 2 DM: a 15 months follow-up study. Indian J Endocrinol Metab.

[9] Li Z, Hao H, Liu J, Li Y, Han W, Mu Y (2017). Mesenchymal stem cell therapy in type 2 diabetes mellitus. Diabetol Metab Syndr.

[10] Pileggi A (2012). Mesenchymal stem cells for the treatment of diabetes. Diabetes..

[11] Wang ZX, Cao JX, Li D, Zhang XY, Liu JL, Li JL (2015). Clinical efficacy of autologous stem cell transplantation for the treatment of patients with type 2 diabetes mellitus: a meta-analysis. Cytotherapy..

[12] Bhansali S, Dutta P, Kumar V, Yadav MK, Jain A, Mudaliar S (2017). Efficacy of autologous bone marrow-derived mesenchymal stem cell and mononuclear cell transplantation in type 2 diabetes mellitus: a randomized, placebo-controlled comparative study. Stem Cells Dev.

[13] Si Y, Zhao Y, Hao H, Liu J, Guo Y, Mu Y (2012). Infusion of mesenchymal stem cells ameliorates hyperglycemia in type 2 diabetic rats: identification of a novel role in improving insulin sensitivity. Diabetes..

[14] Johnson AMF, Olefsky JM (2013). The origins and drivers of insulin resistance. Cell..

[15] Mcnelis JC, Olefsky JM (2014). Macrophages, immunity, and metabolic disease. Immunity..

[16] Olefsky Jerrold M., Glass Christopher K. (2010). Macrophages, Inflammation, and Insulin Resistance. Annual Review of Physiology.

[17] Ji Y, Sun S, Xu A, Bhargava P, Yang L, Lam KSL (2012). Activation of natural killer T cells promotes M2 macrophage polarization in adipose tissue and improves systemic glucose tolerance via interleukin-4 (IL-4)/STAT6 protein signaling axis in obesity. J Biol Chem.

[18] Murray PJ, Wynn TA (2011). Protective and pathogenic functions of macrophage subsets. Nat Rev Immunol.

[19] Odegaard JI, Ricardo-Gonzalez RR, Goforth MH, Morel CR, Subramanian V, Mukundan L (2007). Macrophage-specific PPAR controls alternative activation and improves insulin resistance. Nature..

[20] Xie Z, Hao H, Tong C, Cheng Y, Liu J, Pang Y (2016). Human umbilical cord-derived mesenchymal stem cells elicit macrophages into an anti-inflammatory phenotype to alleviate insulin resistance in type 2 diabetic rats. Stem Cells.

[21] Hao H, Liu J, Shen J, Zhao Y, Liu H, Hou Q (2013). Multiple intravenous infusions of bone marrow mesenchymal stem cells reverse hyperglycemia in experimental type 2 diabetes rats. Biochem Biophys Res Commun.

[22] Hu J, Li C, Wang L, Zhang X, Zhang M, Gao H (2012). Long term effects of the implantation of autologous bone marrow mononuclear cells for type 2 diabetes mellitus. Endocr J.

[23] Liu X, Zheng P, Wang X, Dai G, Cheng H, Zhang Z (2014). A preliminary evaluation of efficacy and safety of Wharton’s jelly mesenchymal stem cell transplantation in patients with type 2 diabetes mellitus. Stem Cell Res Ther.

[24] Issa JP (2005). Optimizing therapy with methylation inhibitors in myelodysplastic syndromes: dose, duration, and patient selection. Nat Clin Pract Oncol.

[25] Jones PA, Taylor SM (1980). Cellular differentiation, cytidine analogs and DNA methylation. Cell..

[26] Momparler RL (2005). Pharmacology of 5-aza-2′-deoxycytidine (decitabine). Semin Hematol.

[27] Issa JPJ, Gharibyan V, Cortes J, Jelinek J, Morris G, Verstovsek S (2005). Phase II study of low-dose decitabine in patients with chronic myelogenous leukemia resistant to imatinib mesylate. J Clin Oncol Off J Am Soc Clin Oncol.

[28] Jabbour E, Issa JP, Garcia-Manero G, Kantarjian H (2008). Evolution of decitabine development: accomplishments, ongoing investigations, and future strategies. Cancer..

[29] Matei D, Fang F, Shen C, Schilder J, Arnold A, Zeng Y (2012). Epigenetic resensitization to platinum in ovarian cancer. Cancer Res.

[30] Chiappinelli KB, Strissel PL, Desrichard A, Li H, Henke C, Akman B (2015). Inhibiting DNA methylation causes an interferon response in cancer via dsRNA including endogenous retroviruses. Cell..

[31] Roulois D, Yau HL, Singhania R, Wang Y, Danesh A, Shen SY (2015). DNA-demethylating agents target colorectal cancer cells by inducing viral mimicry by endogenous transcripts. Cell..

[32] Sánchezabarca LI, Gutierrezcosio S, Santamaría C, Caballerovelazquez T, Blanco B, Herrerosánchez C (2010). Immunomodulatory effect of 5-azacytidine (5-azaC): potential role in the transplantation setting. Blood..

[33] Cao Q, Wang X, Jia L, Mondal AK, Diallo A, Hawkins GA (2014). Inhibiting DNA methylation by 5-aza-2′-deoxycytidine ameliorates atherosclerosis through suppressing macrophage inflammation. Endocrinology..

[34] Kim YS, Kang WS, Kwon JS, Hong MH, Jeong HY, Jeong HC (2014). Protective role of 5-azacytidine on myocardial infarction is associated with modulation of macrophage phenotype and inhibition of fibrosis. J Cell Mol Med.

[35] Thangavel J, Samanta S, Rajasingh S, Barani B, Xuan YT, Dawn B (2015). Epigenetic modifiers reduce inflammation and modulate macrophage phenotype during endotoxemia-induced acute lung injury. J Cell Sci.

[36] Zhang Z, Wang S, Zhou S, Yan X, Wang Y, Chen J (2014). Sulforaphane prevents the development of cardiomyopathy in type 2 diabetic mice probably by reversing oxidative stress-induced inhibition of LKB1/AMPK pathway. J Mol Cell Cardiol.

[37] Wei L, Ming Z, Jian G, Meng ZJ, Zhao LC, Zheng YN (2012). Hypoglycemic effect of protopanaxadiol-type ginsenosides and compound K on type 2 diabetes mice induced by high-fat diet combining with streptozotocin via suppression of hepatic gluconeogenesis. Fitoterapia..

[38] Yin Y, Hao H, Cheng Y, Zang L, Liu J, Gao J, et al. Human umbilical cord-derived mesenchymal stem cells direct macrophage polarization to alleviate pancreatic islets dysfunction in type 2 diabetic mice. Cell Death Dis. 2018;9(7):760. 10.1038/s41419-018-0801-9. 10.1038/s41419-018-0801-9 PMC6037817 29988034

[39] Bieback K, Kern S, Klüter H, Eichler H (2010). Critical parameters for the isolation of mesenchymal stem cells from umbilical cord blood. Stem Cells.

[40] Li W, Zhang Q, Wang M, Wu H, Mao F, Zhang B (2013). Macrophages are involved in the protective role of human umbilical cord-derived stromal cells in renal ischemia–reperfusion injury. Stem Cell Res.

[41] Gordon S, Martinez FO (2010). Alternative activation of macrophages: mechanism and functions. Immunity..

[42] Van Dyken SJ, Locksley RM (2013). Interleukin-4- and interleukin-13-mediated alternatively activated macrophages: roles in homeostasis and disease. Annu Rev Immunol.

[43] Hu J, Wang F, Sun R, Wang Z, Yu X, Wang L (2014). Effect of combined therapy of human Wharton's jelly-derived mesenchymal stem cells from umbilical cord with sitagliptin in type 2 diabetic rats. Endocrine..

[44] Hussien NI, Ebrahim N, Mohammed OM, Sabry D (2017). Combination of obestatin and bone marrow mesenchymal stem cells prevents aggravation of endocrine pancreatic damage in type II diabetic rats. Int J Stem Cells.

[45] Nie J, Zhang Y, Li X, Chen M, Liu C, Han W (2016). DNA demethylating agent decitabine broadens the peripheral T cell receptor repertoire. Oncotarget..

[46] Wang L, Amoozgar Z, Huang J, Saleh MH, Xing D, Orsulic S (2015). Decitabine enhances lymphocyte migration and function and synergizes with CTLA-4 blockade in a murine ovarian cancer model. Cancer Immunol Res.

[47] Wang X, Qiang C, Yu L, Shi H, Xue B, Hang S (2016). Epigenetic regulation of macrophage polarization and inflammation by DNA methylation in obesity. Jci Insight.

[48] Olefsky JM, Glass CK (2010). Macrophages, inflammation, and insulin resistance. Annu Rev Physiol.

[49] Thomas D, Apovian C (2017). Macrophage functions in lean and obese adipose tissue. Metabolism..

[50] Bhattacharjee, Shukla, Yakubenko, Mulya, Kundu (2013). IL-4 and IL-13 employ discrete signaling pathways for target gene expression in alternatively activated monocytes/macrophages. Free Radic Biol Med.

