# Supplementary material for: Decitabine assists umbilical cord-derived mesenchymal stem cells in improving glucose homeostasis by modulating macrophage polarization in type 2 diabetic mice
Source: Stem Cell Res Ther. 2019 Aug 19;10:259. doi: 10.1186/s13287-019-1338-2 (PMC6700792; doi:10.1186/s13287-019-1338-2)
Supplement: Supplementary file 1 — Table S1. Primer sequences of target genes (mice). Figure S1. Induction of the T2D mouse model. The T2D mouse model was induced by a combination of an 8-week HFD and a single intraperitoneal injection of low-dose STZ (100 mg/kg). a Blood glucose was consecutively detected after STZ injection. One week after STZ injection, IPGTT (b) and IPITT (c) were performed to confirm the establishment of the T2D mouse model. For IPITT (c), the results are presented relative to the initial blood glucose levels. The results are presented as the means ± SD. *P < 0.05, **P < 0.01. Figure S2. Identification of UC-MSC characteristics. UC-MSCs were isolated and identified from full-term fetal umbilical cords. a The morphology of UC-MSCs. (B-C): Multilineage differentiation of MSCs: adipocytic differentiation (b) was detected by Oil Red O staining; osteoblastic differentiation (c) was confirmed by Alizarin Red staining; Scale bar 50 μm. (d–f): Immunological phenotypes of MSCs: cultured UC-MSCs consisted of a homogenous mesenchymal population that stained positive for CD90 in 99.76% of cells (d) and CD105 in 99.79% of cells (e) but negative for CD45 (f), which met the international definition of MSCs. Figure S3. Biological characteristics and identification of bone marrow-derived macrophages (BMDMs). BMDMs were isolated and cultured in RMPI-1640 supplemented with 100 ng/ml M-CSF. a The morphology of macrophages. b Flow cytometry analyses showed that the positive rate of F4/80 in bone marrow-derived cells was over 95%. (c, d): M1 macrophage induction, after stimulation with LPS (100 ng/ml) and IFN-γ (50 ng/ml) for 24 h, and the morphology of macrophages (c). The positive rate of CD11c in BMMCs was over 95% (d). Abbreviations: BMDMs, bone marrow-derived macrophages. M-CSF, macrophage colony-stimulating factor. Figure S4. UC-MSCs combined with decitabine did not influence UC-MSCs homing in T2DM mice. UC-MSCs were CM-Dil (red) labeled in advance. Successfully induced type 2 diabetic mice wer [file 13287_2019_1338_MOESM1_ESM.docx]

**Supplementary Figure Legends**

**Supplementary Fig 1. Induction of the T2D mouse model.** The T2D mouse model was induced by a combination of an 8-week HFD and a single intraperitoneal injection of low-dose STZ (100 mg/kg). **a** Blood glucose was consecutively detected after STZ injection. One week after STZ injection, IPGTT (**b**) and IPITT (**c**) were performed to confirm the establishment of the T2D mouse model. For IPITT (**c**), the results are presented relative to the initial blood glucose levels. The results are presented as the means ± SD. **p*<0.05, ***p*<0.01.


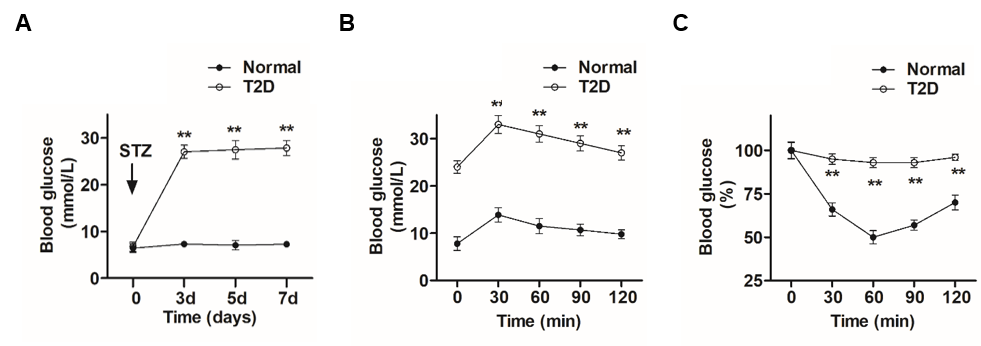


**Supplementary Fig 2. Identification of UC-MSC characteristics.** UC-MSCs were isolated and identiﬁed from full-term fetal umbilical cords. **a** The morphology of UC**-**MSCs. (B-C): Multilineage differentiation of MSCs: adipocytic differentiation (**b**) was detected by Oil Red O staining; osteoblastic differentiation (**c**) was confirmed by Alizarin Red staining; Scale bar: 50 µm. (**d-f**): Immunological phenotypes of MSCs: cultured UC-MSCs consisted of a homogenous mesenchymal population that stained positive for CD90 in 99.76% of cells (**d**) and CD105 in 99.79% of cells (**e**) but negative for CD45 (**f**), which met the international definition of MSCs.

**
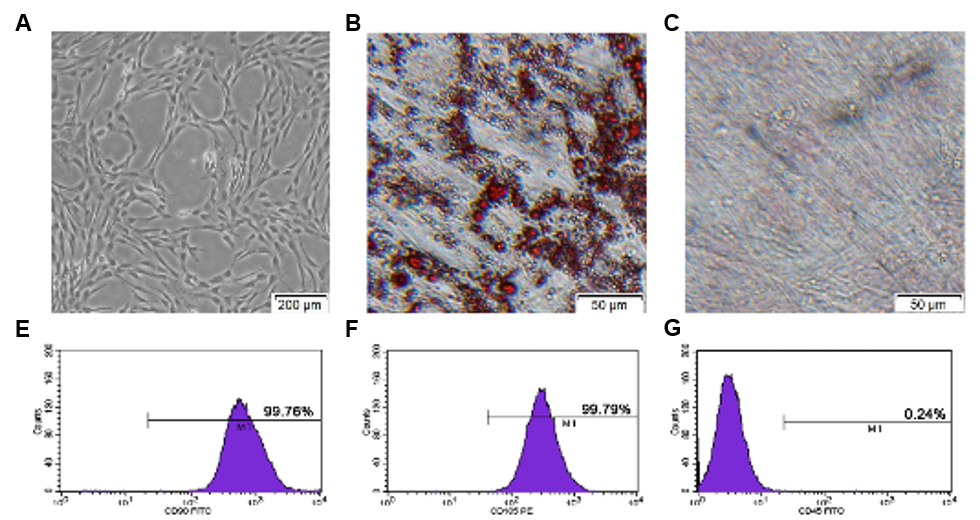
**

**Supplementary Fig 3. Biological characteristics and identification of bone marrow-derived macrophages (BMDMs).** BMDMs were isolated and cultured in RMPI-1640 supplemented with 100 ng/ml M-CSF. **a** The morphology of macrophages. **b** Flow cytometry analyses showed that the positive rate of F4/80 in bone marrow-derived cells was over 95%. (**c, d**): M1 macrophage induction, after stimulation with LPS (100 ng/ml) and IFN-γ (50 ng/ml) for 24 hours, and the morphology of macrophages (**c**). The positive rate of CD11c in BMMCs was over 95% (**d**). Abbreviations: BMDMs, bone marrow-derived macrophages. M-CSF, macrophage colony-stimulating factor.


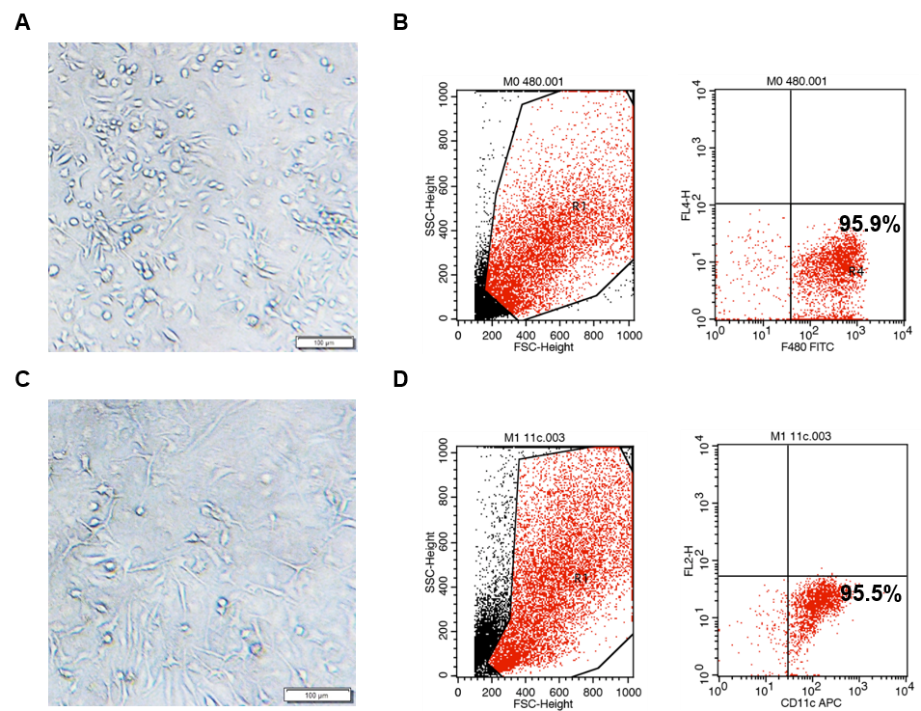


**Supplementary Fig 4. UC-MSCs combined with decitabine did not influence UC-MSCs homing in T2DM mice.** UC-MSCs were CM-Dil (red) labelled in advance. Successfully induced type 2 diabetic mice were treated with UC-MSCs or UC-MSCs combined with decitabine. One week after the infusion, detection of UC-MSCs in the adipose tissue, liver and lung of T2DM recipients using a confocal laser scanning microscope. Scale bar, 100 μm (A) and 50 μm (B). Values are presented as the means ± SD. ns, no significant difference.


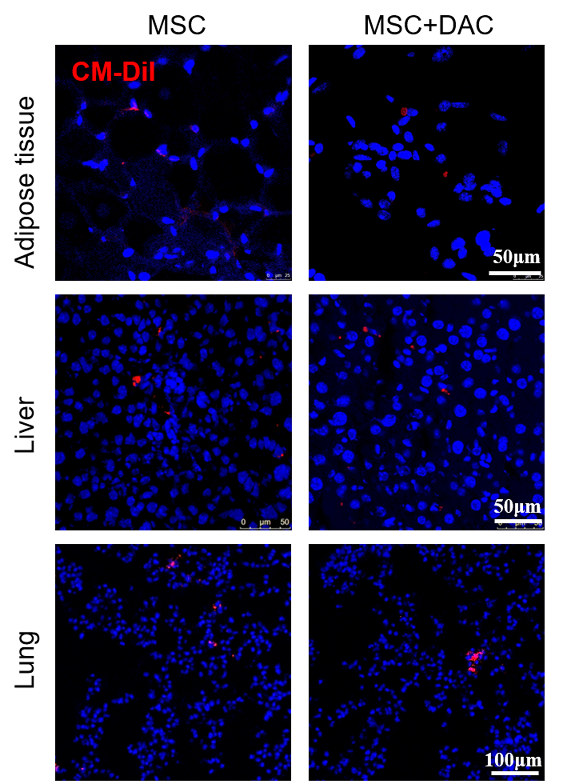


**Supplementary Table 1. Primer sequences of target genes (mice)**

| Genes | Primer sequence (5’-3’) |
| --- | --- |
| β-actin | For: CCAGTTGGTAACAATGCCATG |
|  | Rev: GGCTGTATTCCCCTCCATCG |
| MCP-1 | For: AGGTCCCTGTCATGCTTCTG |
|  | Rev: GCTGCTGGTGATCCTCTTGT |
| TNF-α | For: CCAGACCCTCACACTCAGATC |
|  | Rev: CACTTGGTGGTTTGCTACGAC |
| IL-1β | For: TGGGCCTCAAAGGAAAGAAT |
|  | Rev: CAGGCTTGTGCTCTGCTTGT |
| IL-6 | For: TAGTCCTTCCTACCCCAATTTCC |
|  | Rev: TTGGTCCTTAGCCACTCCTTC |
| IL-4 | For: GGTCTCAACCCCCAGCTAGT |
|  | Rev: GCCGATGATCTCTCTCAAGTGAT |
| IL-10 | For: GCTCTTACTGACTGGCATGAG |
|  | Rev: CGCAGCTCTAGGAGCATGTG |
| Nos2 | For: ACCTTGGTGAAGGGACTGAG |
|  | Rev: TCCGTTCTCTTGCAGTTGAC |
| CD11c | For: ACGTCAGTACAAGGAGATGTTGGA |
|  | Rev: ATCCTATTGCAGAATGCTTCTTTACC |
| CD206 | For: TGATTACGAGCAGTGGAAGC |
|  | Rev: GTTCACCGTAAGCCCAATTT |
| Arg1 | For: AGACCACAGTCTGGCAGTTG |
|  | Rev: CCACCCAAATGACACATAGG |
| TGF-β | For: ATTCCTGGCGTTACCTTGG  Rev: AGCCCTGTATTCCGTCTCCT |
|  |  |
| DNMT1 | For: TGAGGAAGGCTACCTGGCTA |
|  | Rev: ACAACCGTTGGCTTTTTGAG |
| DNMT3a | For: TCGGACCCCGCAACTC |
|  | Rev: CAAGCCCTCGGAAAAGTGC |
| DNMT3b | For: GCCAGACCTTGGAAACCTCA |
|  | Rev: GCTGGCACCCTCTTCTTCAT |
